# Survival benefit of pure dose-dense chemotherapy in breast cancer: a meta-analysis of randomized controlled trials

**DOI:** 10.1186/s12957-018-1424-4

**Published:** 2018-07-14

**Authors:** Wenqi Zhou, Shizhe Chen, Faliang Xu, Xiaohua Zeng

**Affiliations:** 1 Breast Center, Chongqing University Cancer Hospital & Chongqing Cancer Institute & Chongqing Cancer Hospital, Chongqing, 400030 People’s Republic of China; 2 Xiehe Affiliated Hospital of Fujian Medical University, Fuzhou, 350000 Fujian People’s Republic of China

**Keywords:** Dose-dense, Chemotherapy, Breast cancer, Overall survival, Meta-analysis

## Abstract

**Background:**

Dose-dense chemotherapy is a widely accepted regimen for high-risk breast cancer patients. However, conflicting survival benefits of pure dose-dense chemotherapy have been reported in different randomized controlled trials (RCTs). This meta-analysis aimed to further assess the efficacy and safety of pure dose-dense chemotherapy in breast cancer.

**Methods:**

A literature search of electronic databases and websites was performed to identify phase III RCTs reporting the efficacy and toxicity of pure dose-dense chemotherapy. The endpoints of interest were overall survival (OS), disease-free survival (DFS), and toxicities. The hazard ratios (HRs) of death and recurrence and the odds ratios (ORs) of adverse events were estimated and pooled.

**Results:**

Seven studies (five trials) were eligible, encompassing a total of 9851 patients. Patients treated with dose-dense chemotherapy obtained better DFS (HR = 0.83; 95% CI 0.75–0.91; *p* = 0.0001) than those treated with the conventional schedule, while OS benefit of dose-dense chemotherapy was less impressive (HR = 0.86; 95% CI 0.73–1.02; *p* = 0.08). However, significant OS benefit was observed in node-positive patients (HR = 0.77; 95% CI 0.66–0.90; *p* = 0.001). The incidence of anemia, pain, and transaminase elevation was higher in the dose-dense chemotherapy arm.

**Conclusions:**

Dose-dense chemotherapy leads to better prognosis; these findings suggest that it may be a potentially preferred treatment for breast cancer patients, particularly for women with lymph node involvement. However, more RCTs are warranted to better define the best candidates for dose-dense chemotherapy.

## Background

Breast cancer is the most commonly diagnosed cancer and the second leading cause of cancer death among women in the USA [1]. Although adjuvant chemotherapy confers about a one-third reduction for 10-year risk of death from breast cancer [2], a large number of patients will suffer from recurrence and breast cancer-related death. Thus, to further optimize prognoses of breast cancer patients with elevated recurrence risk, different approaches have been taken to improve the efficacy of chemotherapy, including the addition of new drugs or modifications of drug delivery.

According to the Norton-Simon hypothesis [3] and the Gomepertzian growth pattern [4], delivering drugs at shorter intervals may maximize the possibility of eradicating tumor cells by shortening the time for tumor regression between treatments. Dose-dense chemotherapy, in which drugs are delivered with shorter interval between treatments, is a widely accepted regimen for high-risk breast cancer patients [5]. Even so, treatment guidelines vary from Europe [6] to America [5]. According to the St. Gallen International Breast Cancer Consensus in 2017 [6], there were no clear recommendations for dose-dense chemotherapy, and less than half of the attendees thought that dose-dense regimens should be preferred in triple-negative patients. Furthermore, the few existing studies based on pure dose-dense chemotherapy, in which drugs were administered at shorter intervals with the same cycles and doses of conventional regimen, have reported conflicting results. The CALGB 9741 [7] and GIM2 [8] trials demonstrated that dose-dense chemotherapy significantly improved disease-free survival (DFS) and overall survival (OS), while no survival benefit of dose-dense chemotherapy was observed in the MIG-1 [9] and TACT2 [10] trials. Meta-analyses have shown that dose-dense chemotherapy produces a significant improvement in DFS, especially in patients with negative hormone receptor, while the results of OS were controversial [11–13]. However, few of them were based on pure dose-dense trials, and thus, the real benefit of the increase in dose density cannot be assessed appropriately due to the introduction of confounding factors. Furthermore, none of the previous meta-analyses included the new results of the TACT2 trial. Therefore, this updated meta-analysis was performed to further investigate the efficacy and toxicity of pure dose-dense chemotherapy.

## Methods

This meta-analysis was performed in accordance with the recommendation outlined in the Preferred Reporting Items for Systematic Reviews and Meta-Analyses (PRISMA) statement [14]. A literature search was performed using the databases of PubMed/MEDLINE, Cochrane library, EMBASE through 1 September 2017. In addition, the ASCO, SABCS, and ESMO Meeting websites were scrutinized. The search strategy was developed using the following terms: (breast cancer OR breast tumor OR breast neoplasms OR breast carcinoma) and (drug therapy OR chemotherapy) and ((dose dense) OR accelerat* OR (14 days) OR (2 weeks) OR biweekly OR weekly OR (2 weekly)) and (random* OR prospective*).

### Selection criteria

This meta-analysis was based on phase III RCTs in which the dose-dense regimen of the experimental arm was narrowly defined as delivering drugs over a shorter interval with the same cycle and dosage of the conventional schedule in the control arm. Full papers and conference abstracts providing sufficient data were eligible. Studies that included metastatic breast cancer patients and studies based on impure dose-dense regimens (with different type or dosage of drugs) were ineligible. In addition, studies without outcomes of interest were excluded.

### Quality assessment

The methodological quality of the eligible studies was independently assessed by two reviewers using the Cochrane risk-of-bias tool, which consists of the following domains of bias: selection bias, performance bias, detection bias, attrition bias, and reporting bias [15, 16]. Disagreements were resolved through discussion and consensus.

### Data extraction

A standardized Excel form was used to extract data from eligible studies, including first author, year of publication, sample size, inclusion criteria, chemotherapy regimen, and median follow-up. The primary endpoint was OS (measured from randomization until death from any cause); other outcomes of interest were DFS (measured from randomization until local recurrence, distant relapse, or death without relapse, whichever occurred first) and incidence of grade 3 to 5 toxicities. The hazard ratios (HRs) and variances of time-to-event data were extracted from the original studies or were estimated as described by Parmar et al. [17] and Tierney et al. [18].

### Statistical analysis

Hazard ratios (HRs) and odds ratios (ORs) were calculated to compare time to event outcomes and dichotomous data, respectively. An HR or OR less than one favored the dose-dense chemotherapy arm. The meta-analyses of outcomes were based on a fixed-effect model, except the outcomes with significant heterogeneity, for which a random-effect model was used.

Heterogeneity was quantified using the inconsistency index (*I*^2^) and the *p* value of the *χ*^2^ test. Significant heterogeneity was considered to exist for *p* values less than 0.1 or *I*^2^ greater than 50%. Subgroup analyses were conducted according to hormone receptor status of the tumors and the inclusion criteria of the studies to assess potential contributions to outcomes. Publication bias was evaluated by funnel plots and Egger’s test [19]. The meta-analysis was performed using RevMan version 5.3.

## Results

### Study selection

**Fig. 1:**
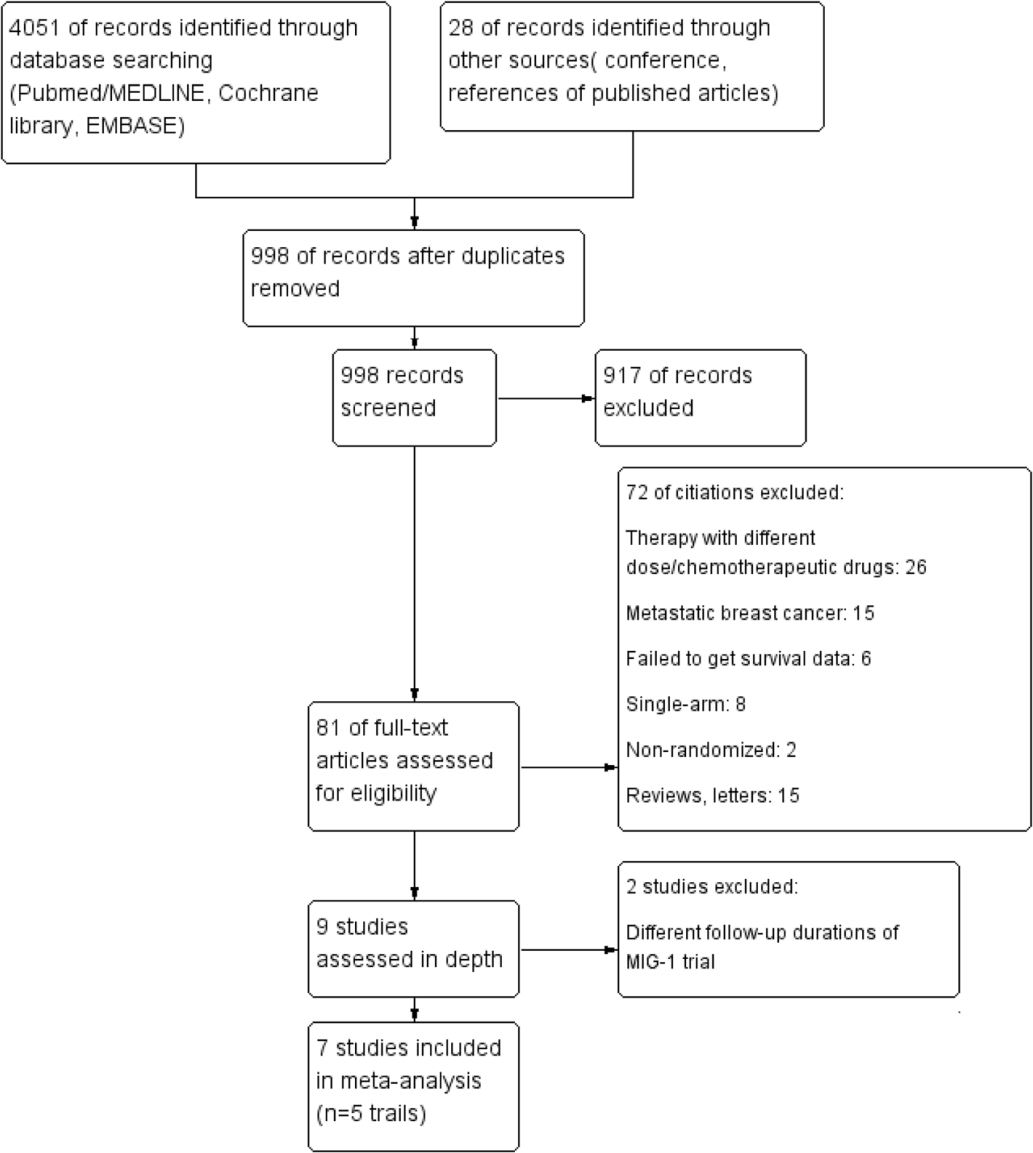
Flowchart of the study selection process and exclusion criteria

According to the research strategy, a total of 4079 studies were retrieved, of which 3081 studies were removed owing to duplication or overlap using Endnote software. Another 917 studies were excluded by screening the titles and abstracts. After reading the remaining 81 full-text articles, 74 studies were excluded. Ultimately, 7 studies [7–10, 20–22] based on 5 phase III RCTs that compared pure dose-dense chemotherapy with conventional chemotherapy were included. Figure 1 shows the details of the study selection process and the exclusion criteria.

### Characteristics of eligible studies

The characteristics of the included studies are listed in Table 1. A total of 9851 node-positive or high-risk node-negative patients were included in this meta-analysis. Four studies (three trials) [9, 10, 20, 21] were based on anthracycline, while the other three studies (two trials) [7, 8, 22] were based on anthracycline and taxane. Among the five included trials, survival data of the CALGB 9741 trial and MIG-1 trial were updated in abstract forms at the San Antonio Breast Cancer Symposium in 2005 [22] and the European Society for Medical Oncology in 2016 [21], respectively. The risk of bias for each study is reported in Table 2. TACT2 trial [10] was judged as high risk for reporting bias due to incomplete reporting of the DFS outcome.

**Table 1:** Characteristics of included studies

Study	*N*	Patients	Treatment	MF	DFS HR(95%CI) DD vs Con	OS HR(95%CI) DD vs Con
Baldini 2003	150	IIIA/B	dd(CEF → CMF/CEF) CEF → CMF/CEF	5 years	0.77(0.47–1.26)	0.87(0.49–1.53)
CALGB 9741						
(1)Citron 2003	2005	T0–3, N1–2, M0	dd(A → P → C) A → P → C dd(AC → P) AC → P	36 months	0.74(0.59–0.93)	0.69(050–0.93)
(2)Hudis 2005				69 months	0.80(0.67–0.96)	0.85(0.68–1.05)
MIG-1						
(1)Venturini 2005	1214	pN+(≤ 10); pN− and high risk	ddFEC FEC	10.4 years	0.88(0.71–1.08)	0.87(0.67–1.13)
(2)Giraudi 2016				15.8 years	0.90(0.77–1.05)	0.89(0.72–1.09)
GIM2	2091	pN+(≥ 1)	dd(EC → P) EC → P dd(FEC → P) FEC → P	7 years	0.77(0.65–0.92)	0.65(0.51–0.84)
Mastro 2015						
TACT2	4391	≥ 18 years; pN+; pN− and high risk (T0–3,N0–2,M0)	ddE → CMF E → CMF ddE → X E → X	85.6 months	NA	1.04(0.88–1.21)
Cameron 2017						

*N* number of patients, *MF* median follow-up, *DFS* disease-free survival, *OS* overall survival, *HR* hazard ratio, *CI* confidence interval, *DD* dose-dense chemotherapy, *Con* conventional chemotherapy, *NA* not available, *CEF* cyclophosphamide + epirubicin + 5-fluorouracil; CMF, cyclophosphamide + methotrexate + 5-fluorouracil, *A* doxorubicin, *P* paclitaxel, *C* cyclophosphamide, *AC* doxorubicin + cyclophosphamide, *FEC* 5-fluorouracil + epirubicin + cyclophosphamide, *X* capecitabine

**Table 2:** Risk of bias summary for each included study

Study	Selection bias	Performance bias	Detection bias	Attrition bias	Reporting bias	Other bias
Cameron 2017 (TACT2)	Low	Low	Low	Low	High	Low
Baldini 2003	Low	Low	Low	Low	Low	Low
Citron 2003/Hudis 2005 (CALGB 9741)	Unclear	Low	Low	Low	Low	Low
Venturini 2005/Giraudi 2016 (MIG-1)	Low	Low	Low	Low	Low	Low
Mastro 2015 (GIM2)	Low	Low	Low	Low	Low	low

### Overall survival

**Fig. 2:**
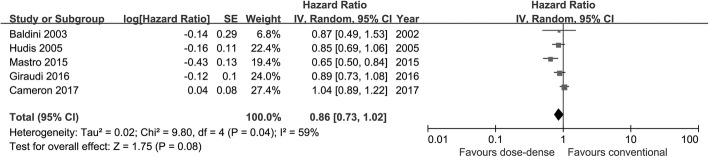
Forest plot of hazard ratios comparing overall survival of patients treated with dose-dense chemotherapy versus that of those treated with conventional chemotherapy

**Fig. 3:**
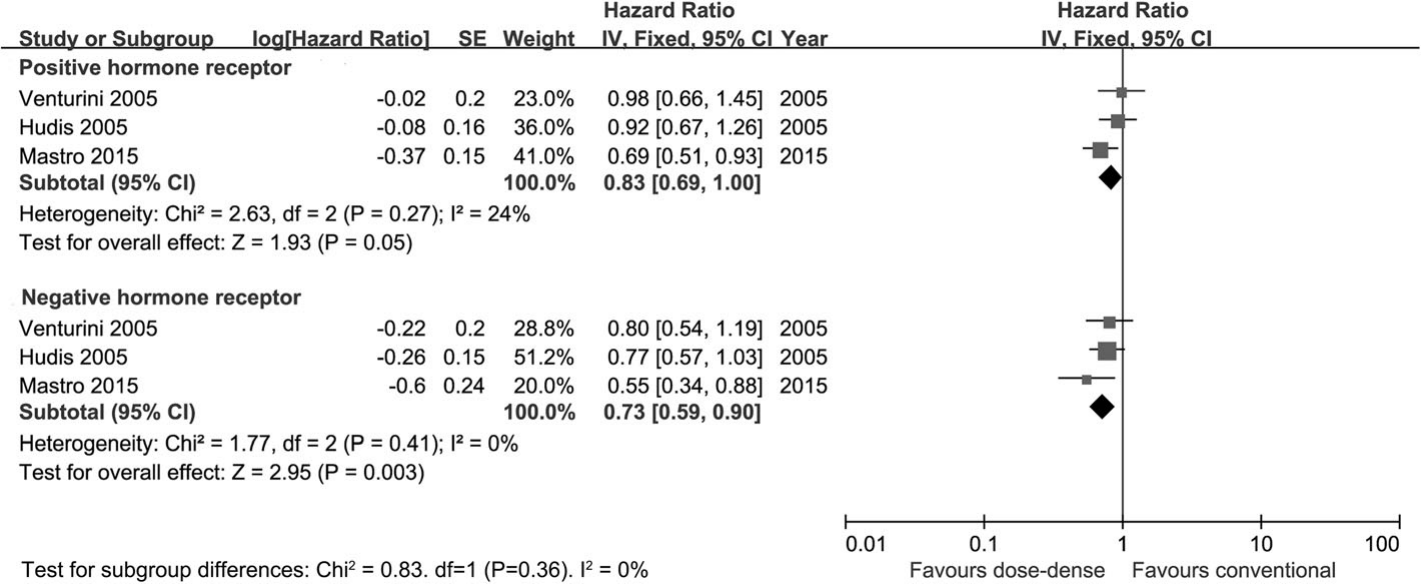
Forest plot of hazard ratios comparing overall survival of patients treated with dose-dense chemotherapy versus that of those treated with conventional chemotherapy according to tumor hormone receptor status

A total of 9731 patients were included in the OS meta-analysis. Updated abstracts [21, 22] of two eligible trials [7, 9] were included in this meta-analysis. Patients in the dose-dense arm failed to obtain a significant OS benefit compared with those in the conventional arm (HR = 0.86; 95%CI 0.73–1.02; *p* = 0.08). A random effect model was used due to the high heterogeneity among studies (*I*^2^ = 59%) (Fig. 2). According to the subgroup analysis based on hormone receptor status, dose-dense chemotherapy produced significant OS benefit in patients with negative hormone receptor status (HR = 0.73; 95%CI 0.59–0.90; *p* = 0.003; *I*^2^ = 0%), but not in hormone receptor-positive patients (HR = 0.83; 95% CI 0.69–1.00; *p* = 0.05; *I*^2^ = 0%). However, there was no sign of interaction between survival benefit of the dose-dense regimen and hormone receptor status (interaction test, *p* = 0.36). Figure 3 illustrates the analysis according to hormone receptor status.

### Disease-free survival

**Fig. 4:**
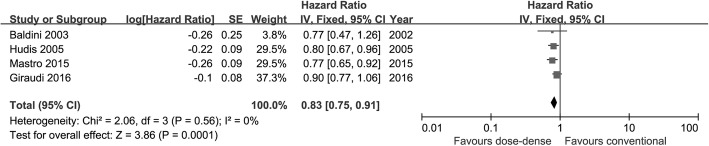
Forest plot of hazard ratios comparing disease-free survival of patients treated with dose-dense chemotherapy versus that of those treated with conventional chemotherapy

The meta-analysis of DFS covered 5340 patients. According to the result, DFS was significantly improved in the dose-dense arm (HR = 0.83; 95% CI 0.75–0.91; *p* = 0.0001), with no heterogeneity (*I*^2^ = 0%) (Fig. 4). Considering the different hormone receptor status, dose-dense chemotherapy conferred a significant improvement in DFS in patients with hormone receptor-negative tumor (HR = 0.74; 95%CI 0.62–0.89; *p* = 0.001; *I*^2^ = 0%), while patients with hormone receptor-positive tumor obtained no significant DFS benefit (*p* = 0.53) (interaction test, *p* = 0.20).

### Toxicities

The incidences of grade 3 to 5 neutropenia (OR = 0.14; 95% CI 0.09–0.24; *p* < 0.0001), leukopenia (OR = 0.39; 95% CI 0.28–0.55; *p* < 0.0001), and neuropathy (OR = 0.72; 95% CI 0.54–0.97; *p* = 0.03) were significantly lower in the dose-dense arm than those in the conventional arm. However, pooled analyses demonstrated that dose-dense chemotherapy significantly increased the incidences of grade 3 to 5 anemia (OR = 4.08; 95% CI 0.67–9.99; *p* = 0.002), pain (OR = 1.67; 95% CI 1.24–2.55; *p* = 0.0007), and transaminase elevation (OR = 3.71; 95% CI 1.50–9.17; *p* = 0.005) compared with the conventional regimen. There was no difference between dose-dense and conventional chemotherapy in terms of thrombocytopenia, asthenia, diarrhea, stomatitis, nausea/vomiting, and infection. The details are shown in Table 3.

**Table 3:** Meta-analysis of toxicities comparing dose-dense chemotherapy versus conventional chemotherapy

Toxicity (grade 3 to 5)	*N*	OR (95%CI)	*I*^2^ (%)	*p* value
Anemia	7379	4.08 [1.67, 9.99]	0	0.002
Neutropenia	6049	0.14 [0.09, 0.24]	87	< 0.0001
Leukopenia	5407	0.39 [0.28, 0.55]	26	< 0.0001
Thrombocytopenia	7379	1.10 [0.47, 2.54]	0	0.83
Asthenia	5271	1.28 [0.93, 1.75]	0	0.13
Diarrhea	7233	1.16 [0.73, 1.86]	0	0.53
Pain	7379	1.67 [1.24, 2.25]	0	0.0007
Stomatitis	7379	1.37 [0.88, 2.15]	0	0.17
Nausea/vomiting	7379	1.18 [0.97, 1.42]	0	0.09
Neuropathy	7233	0.72 [0.54, 0.97]	0	0.03
Transaminase elevation	5271	3.71 [1.50, 9.17]	0	0.005
Infection	5271	0.86 [0.62, 1.19]	0	0.35

*N* number of patients, *OR* odds ratio, *CI* confidence interval

### Heterogeneity

**Fig. 5:**
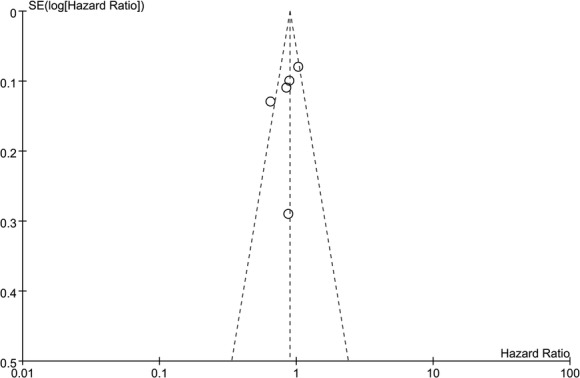
Funnel plot of overall survival in all eligible trials for the visual detection of systematic publication bias and small study effects

**Fig. 6:**
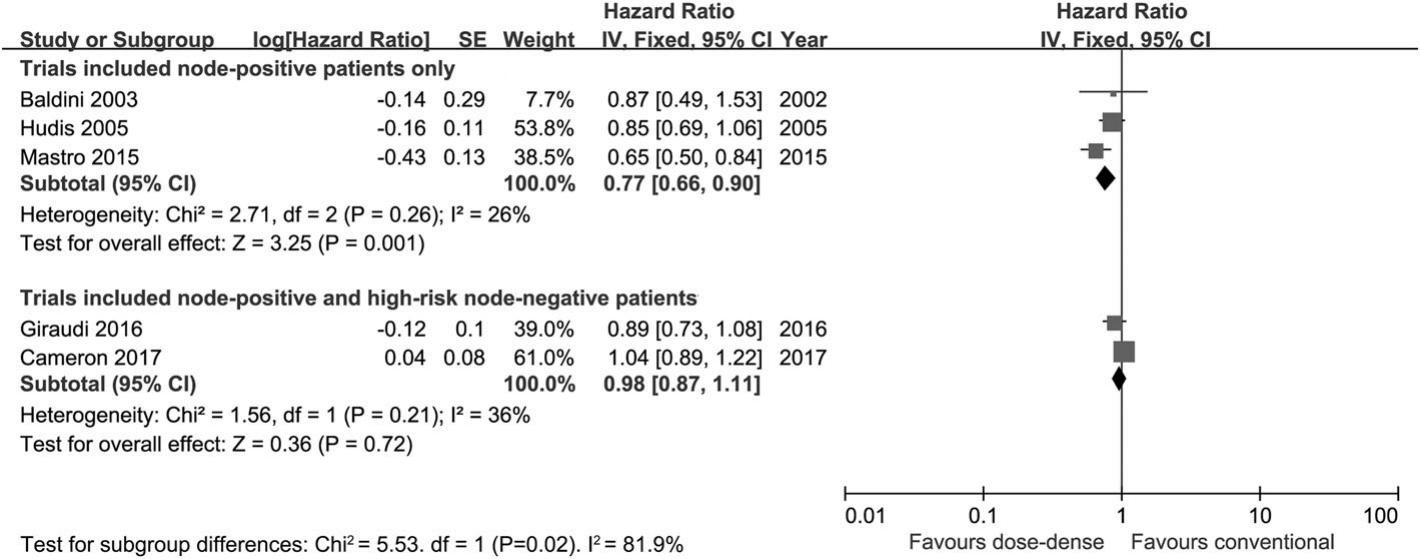
Forest plot of hazard ratios comparing overall survival of patients treated with dose-dense chemotherapy versus that of those treated with conventional chemotherapy in trials including node-positive patients only and in trials including node-positive/high-risk node-negative patients

As previously mentioned, there was high heterogeneity in the pooled analysis of OS (*I*^2^ = 59%). Neither the funnel plot (Fig. 5) nor Egger’s test (*p* = 0.729) indicated significant publication bias. To explore the between-study heterogeneity, a subgroup analysis was performed based on the characteristics of the included patients. The pooled analysis of three studies in which eligible patients all had nodal involvement demonstrated significantly better OS in the dose-dense arm (HR = 0.77; 95% CI 0.66–0.90; *p* = 0.001; *I*^2^ = 26%). While the pooled results from the other two studies, which included both node-positive and high-risk node-negative patients, failed to show significant benefit (HR = 0.98; 95% CI 0.87–1.11; *p* = 0.72; *I*^2^ = 36%). In addition, there was a significant interaction between OS benefit of dose-dense chemotherapy and patient characteristics (*p* = 0.02; see Fig. 6).

## Discussion

As a newly updated meta-analysis based on phase III RCTs regarding the efficacy and safety of pure dose-dense chemotherapy, the pooled results demonstrated a 17% reduction in risk of recurrence and a 14% reduction in risk of death, though the OS benefit was less obvious. A possible explanation for the lack of significant OS benefit may be the insufficient follow-up duration, which prevents the real impact of the dose-dense regimen to be verified. Additionally, the specific agents and total dose in eligible studies may vary from state-of-the-art regimens, especially those conducted in early years. According to the subgroup analysis by inclusion criterion, the pooled analysis of studies based on patients with lymph node involvement showed a significant OS benefit in the dose-dense arm, while studies including patients without lymph node involvement did not. Furthermore, the interaction test showed significant evidence of interaction between dose-dense benefit and patient selection (interaction test, *p* = 0.02). Therefore, the heterogeneity may largely be driven by the different inclusion criteria, especially the nodal status of patients. This was supported by the AGO trial [23], which demonstrated a more pronounced benefit of intense dose-dense chemotherapy among patients with 10 or more involved lymph nodes (HR = 0.64; *p* = 0.0012). A similar effect was also observed in the MIG-1 trial [9]. In this study, the OS benefit of dose-dense chemotherapy seemed to be restricted to patients with lymph node involvement, but there were insufficient primary studies to further assess the survival benefit of dose-dense chemotherapy in patients with different nodal status.

With the support of pegfilgrastim, the incidence of neutropenia was significantly reduced in the dose-dense arm. The high heterogeneity (*I*^2^ = 87%) may be due to higher incidence of neutropenia resulting from FEC-P (fluorouracil, epirubicin, cyclophosphamide followed by paclitaxel) in the GIM2 trial [8]. However, dose-dense schedules inevitably increased the risk of anemia, pain, and transaminase elevation. There was insufficient data for this meta-analysis to assess treatment-induced amenorrhea. The TACT2 trial [10] demonstrated that the risk of permanently discontinued menstruation did not differ between dose-dense and conventional chemotherapy. In addition, a pooled analysis focusing on premenopausal patients also confirmed no increased risk of amenorrhea with dose-dense chemotherapy [24]. Hence, dose-dense chemotherapy seems to be an effective and tolerable treatment choice for breast cancer patients.

Similar meta-analyses performed by Bonilla et al. [11] and Petrelli et al. [13] both suggested that dose-dense chemotherapy was associated with improved OS and DFS, especially in hormone receptor-negative patients. However, it should be noted that both of these meta-analyses included trials with impure study design. Therefore, the interpretability of these results was confounded by the variety of dose intensity, type of drug, and cycle number of chemotherapy between dose-dense and control groups. To conduct a true test of the dose-dense concept without confounders, we narrowly defined the dose-dense schedule. Thus, metronomic chemotherapy, which is a variation of the dose-dense schedule whereby drugs are administered at lower doses and shorter intervals [25], is ineligible for this meta-analysis. As evaluated in the E1199 [26] and S0221 [27] trials, metronomic chemotherapy always represents an intense dose-dense schedule. Unlike these studies, Duarte et al. [12] performed a meta-analysis based on pure dose-dense regimens and reported a similar finding as our study, although they did not include the results of the GIM2 trial [8], the TACT2 trial [10], or the updated result of the MIG1 trial [21].

Therefore, this newest updated meta-analysis further confirms that pure dose-dense chemotherapy leads to prolonged DFS and highlights a significant OS benefit in node-positive patients. Despite the significant improvement in OS among hormone receptor-negative patients treated with dose-dense chemotherapy, there was no sign of interaction between dose-dense benefit and hormone receptor status (interaction test, *p* = 0.36). Thus, the subgroup analysis should be interpreted cautiously, and the greater efficacy of dose-dense chemotherapy in hormone receptor-negative patients elucidated in previous studies may need further investigation. To our knowledge, an EBCTCG meta-analysis reported at the 40th San Antonio Breast Cancer Symposium at an oral session revealed significant reductions in DFS and 10-year breast cancer mortality with dose-dense chemotherapy, which, together with our results, provides further evidence of the efficacy and safety of dose-dense chemotherapy [28].

There are some limitations of this meta-analysis that need to be addressed. First, the limited number of eligible studies may contribute to unstable results, which may be influenced by unpublished data and further studies, even though there was no sign of significant publication bias according to the funnel plot and Egger’s test. Second, this is a meta-analysis based on published literature instead of individual patients, inevitable bias resulting from different study designs may lead to a less reliable result. Third, the chemotherapy regimens of the studies were different, and therefore, it is unclear whether the benefit of dose-dense chemotherapy was derived from taxane or anthracycline. Therefore, to gain further understanding of the survival benefit of dose-dense chemotherapy and to identify subgroups of patients who could gain significant benefit from a dose-dense schedule, more RCTs with pure dose-dense designs and longer follow-ups are warranted.

## Conclusion

This study demonstrates that dose-dense chemotherapy leads to improved DFS and highlights a significant OS benefit in node-positive patients. Although limitations exist, this meta-analysis provides further evidence of the dominance and manageable toxicities of dose-dense chemotherapy. It may be a potential preferred treatment for breast cancer patients, particularly for women with lymph node involvement. However, further investigations are needed to better define specific groups of patients who may derive greater benefit from dose-dense chemotherapy.
